# The negotiation of the sick role: general practitioners’ classification of patients with medically unexplained symptoms

**DOI:** 10.1111/j.1467-9566.2011.01448.x

**Published:** 2012-03-05

**Authors:** Nanna Mik-Meyer, Anne Roelsgaard Obling

**Affiliations:** Department of Organisation, Copenhagen Business SchoolFrederiksberg, Denmark

**Keywords:** medically unexplained symptoms, general practitioner, sick role, classification, legitimacy

## Abstract

In encounters between general practitioners (GPs) and patients with medically unexplained symptoms (MUS), the negotiation of the sick role is a social process. In this process, GPs not only use traditional biomedical diagnostic tools but also rely on their own opinions and evaluations of a patient’s particular circumstances in deciding whether that patient is legitimately sick. The doctor is thus a gatekeeper of legitimacy. This article presents results from a qualitative interview study conducted in Denmark with GPs concerning their approach to patients with MUS. We employ a symbolic interaction approach that pays special attention to the external validation of the sick role, making GPs’ accounts of such patients particularly relevant. One of the article’s main findings is that GPs’ criteria for judging the legitimacy of claims by those patients that present with MUS are influenced by the extent to which GPs are able to constitute these patients as people with social problems and problematic personality traits.

## Introduction

In western countries, there are growing numbers of people who report pain throughout the body that cannot be medically diagnosed (Ring *et al.* 2005). The reported pain of these individuals cannot be fully explained by pathology: no physiological tests correlate with the reported symptoms of the patients. This is a patient group that tests the credibility of doctors and the legitimacy of patients: doctors risk professional credibility for their failure to diagnose certain patients’ problems while patients who have symptoms whose causes cannot be diagnosed feel the shame of being illegitimately sick – that is, they are deviant cases for whom a classification rule cannot be applied, despite the claims that they make. These are patients who, in Jutel’s (2010) words, ‘feel poorly, but for whom no medical explanation can be given’ (2010: 230).

In the sociological literature, such deviant and illegitimate patient cases are often characterised as suffering from ‘medically unexplained symptoms (MUS)’ (Bendelow 2009: 57). This is an overarching category incorporating a variety of different conditions such as chronic pain, stress, milder forms of depression and contested illnesses, including fibromyalgia, chemical intolerance, irritable bowel syndrome and chronic fatigue syndrome. Although these illnesses have little in common and this patient group is by no means homogenous, these patients do fall into a particular (residual) category in the eyes of general practitioners (GPs). The patients repeatedly consult their GPs over physical pain that cannot be diagnosed medically. However, in addition to this common feature mentioned by GPs, we note that the acronym MUS is, like any other category, constitutive in nature (see Hacking 1986, Jenkins 2000). As a container category, MUS both contains individual symptoms and complaints of patients and works as a reductionist label that organises different patients into a unitary group. As an unintended consequence of this classification, the MUS label produces and stabilises the expectations of the patients in this group. Here, we focus on that act of classification – in medical terms, the diagnostic trajectory.

This article presents the results from a research project conducted in Denmark in 2008–2009 that focused on patients with MUS (Mik-Meyer 2010, 2011). In the article we show how GPs recognise this patient group, bearing in mind that GPs focus their professional attention as much on the suffering of the patients as on the medical classification of illness. As already demonstrated by Parsons (1951, 1978) and by Balint (1964), the role of a GP is to both address patients’ subjective distress and to diagnose illness through a physical examination. When GPs attempt to find out what is the matter with someone, they must attempt to attribute symptoms to causes and make a diagnosis. This diagnostic process involves linking different symptoms with each other so that eventually the combination of symptoms can be labelled as a disease or a pattern of illness and hence given an explanation.

Patients with MUS, however, make the completion of these tasks difficult. Many of the illnesses in the broad MUS category fall outside the scope of biomedical observation. When people have symptoms that fall outside clear-cut medical diagnoses, doctors tend to either classify the symptoms as psychological in nature or ignore the patients’ physical symptoms altogether (see Sabo *et al.* 2000). Hence, patients with MUS challenge GPs’ traditional approach to diagnosis and intervention and what it takes to be a ‘legitimate’ patient in a context constituted by medical credibility. Credibility refers to a GP’s ability to create an explanatory framework that categorises patients in a specific sick role through the legitimisation of their complaint, even when a medical diagnosis is absent.

This article presumes that when MUS patients turn to GPs for assistance they risk being classified as illegitimate; in turn, in the patients’ eyes, the GPs can be seen to be lacking in credibility as medical practitioners if they cannot state what is wrong with them. This article provides a sociological insight into contemporary understandings of ‘the sick role’ (Parsons 1951: 436) when the patients in question are defined by the lack of a clear-cut medical diagnosis. We also demonstrate how GPs classify symptoms described by MUS patients and create opportunities to treat this group of people as legitimately sick individuals. In this article we address these questions by firstly, examining how different explanatory models reported by GPs relate to their construction of what it is to be a legitimate patient. Secondly, we focus upon how GPs’ struggle to define ‘legitimate patients’ relates to the (problematic) socioeconomic status, general life stories and personality traits of patients with MUS. In our present study, however, we exclusively focus on the GPs’ point of view.

## Classification of illness

There has been much research on the tools of classification in biomedicine (see Bowker and Starr 1999) and how diagnostic practices can be a potent way to create a social order for medicine, the patient and the doctor. In other words, diagnosis structures become a reality for individuals. Jutel (2009) writes how ‘being diagnosed gives permission to be ill. What was previously a complaint is now a disease’ (2009: 278). But what happens when doctors cannot give such permission because there is no clear correspondence between the patients’ complaints and physiological function? Previous research problematises the dichotomy of illness and disease (see Mol 2002) and questions, for example, the utility of classifying illness narratives as either fact or fiction (Bury 2001). Recently, Michailakis and Schirmer (2010) addressed the distinction between being diagnosed and considered to be ill as a medical matter and being so diagnosed as a political matter. This focus follows Parsons’ (1978) original work, which posited that society has a functional interest in minimising illnesses because the sick role effectively inhibits the fulfilment of all other social roles.

Parsons’ (1951, 1978) initial thoughts on the institutional expectations of the sick role thus inspired this article. We focus on GPs’ classification of patients with a similarly strong focus on how the sick role is merged with society’s expectations of its citizens. We do not depart from a dualistic approach that distinguishes between (factual) disease and (fictional) illness narratives of patients. We address the ways in which GPs approach patients who hold the sick role in cases where the patients challenge the ability of the GPs to offer a clear-cut diagnosis. We try to understand how GPs primarily attend to the (problematic) social situation and (problematic) personality of patients with MUS by recognising patients with MUS as legitimate patients and thereby entering them into the sick role. The sick role gives moral legitimacy to the claim that patients cannot perform normal responsibilities and hence that this ‘condition’ prevents them from performing other social roles (see Parsons 1978: 436).

A number of recent empirical studies have examined patients living without a diagnosis, including patients with MUS, and thereby brought prominence to the interdependent relation between the patient, the doctor and the reported complaint. Nettleton (2006), for example, shows how 18 neurology outpatients in England needed (but were not given) permission to be sick. Wilemana *et al.* (2002) explore GPs’ attitudes towards the management of patients that have MUS in primary care consultation. Focusing on the relationship between doctors and patients as well as on problems of control and authority in consultations, the authors discuss the need for more training for GPs on how to manage patients with MUS. In a study on diagnosing depression in primary care, McPherson and Armstrong (2009) show how doctors struggle at first to identify certain patients but then begin to construct a category for such patients that is characterised by non-medical features – for example, by deviant features such as emotional difficulties or manipulative tendencies. Werner and Malterud (2003) use patient experiences in primary care to explore what it takes to be a legitimate patient in the eyes of a doctor when a biomedical diagnosis is unclear (in their terminology ‘a credible patient’). Similarly, Gill *et al.* (2010) focus on how patients take an active part in the interpretation of their own symptoms.

Classification, while it occurs in the context of the medical consultation, is a complex embedded institutional process. Other studies have addressed how the medical system and the public in general deal with patients with MUS by analysing, for example, the documents of support groups and health professionals that deal with fibromyalgia syndrome (Madden and Sim 2006), patient associations and media portrayal of Morgellons (Fair 2010), Internet newsgroup postings and public debates on chronic fatigue syndrome and multiple chemical sensitivity (Dumit 2006), encounters between genetic counsellors and clients ‘without a label’ (diagnosis) (Brookes-Howell 2006) and audio-recorded consultations between GPs and patients with medically unexplained physical syndrome (Ring *et al.* 2005). However, none of these studies addressed how MUS influences the GPs’ classification and recognition of legitimate patients.

## Theoretical framework

This article is inspired by a theoretical approach to the negotiation of the sick role that relates to GPs’ ideas of what it takes to be a legitimate patient. Research into how symptoms are recognised and classified by GPs is affected not only by medical discourse but also by the different social contexts in which the negotiation of the sick role takes place. For this reason, we employ a theoretical approach that takes into account the different contexts in which identity construction (Goffman 1990a, Mead 1934) and negotiation of dominating categories take place.

The explanatory models provided by doctors become highly relevant when we take a symbolic interaction approach (Goffman 1990b, Jenkins 1996, Mead 1934) to the construction of a legitimate sick role. The symbolic interaction approach pays particular attention to the external validation of identities (see, for example, the ‘generalised other’ in Mead 1934: 151), in our case, the GPs’ explanations of the legitimacy of the sick role. Doctors’ external classifications are always related to issues of identification and identity (Jenkins 1996: 113, 120–1). Classification practices are here assumed to influence not only how GPs work and think but also how patients with MUS are perceived and valued in the doctor–patient relationship. The external categorisation performed by GPs gives information about the boundaries around what can be said to constitute a narrative of complaint and grounds the definition of the medical situation.

Shilling (2003) proposes the term ‘the body project’ to emphasise that there is a tendency in our modern, uncertain environment to see the body as ‘in a process of becoming; a *project* which should be worked at and accomplished as part of an *individual’s* identity’ (2003: 4; emphasis in original). It seems reasonable to suggest that, in regard to the categorisation of patients with MUS, the body can be seen as an important workable project. The bodies of patients with MUS are examples of ‘malleable entities, which can be shaped and honed by the vigilance and hard work of their owners’ (2003: 5). Patients with MUS must work laboriously to make their symptoms socially visible, real and physically present (Werner and Malterud 2003). These people, however, cannot do this work alone. Patients with MUS are in strong need of others (Mead 1934), such as GPs, to define their bodies as entities that need repair and, hence, qualify them for the sick role.

A sociological focus on the process in which deviant bodies are repaired in medicine is not new (see Parsons 1951, 1978). As classic work has shown, medicine has a normalising function (see Canguilhem 1989, Foucault 1977). Medicine restores defective organs to health and corrects bodily dysfunctions. Our question, however, is the following: what does medicine actually normalise for patients with MUS? According to the scholars just mentioned, medicine not only restores and repairs parts of the body that are malfunctioning (for example, a broken leg or an infected blood vessel); medicine also seeks to restore patients’ abnormalities that go beyond pathological illness and seeks to deal with norms that define ‘normal’ health in the surrounding society and its institutions. Doctors can in this way be seen as ‘moral entrepreneurs’ (Becker 1997: 147) because they legitimise and label illness.

In our empirical analysis we show how this insight becomes relevant when the GPs, influenced by common sense psychology, repeatedly transmute patients with MUS into objects of inspection and discourse set outside a traditional biomedical vocabulary (Balint 1964, Rose 1985, 1999a). This common sense perspective includes ideas about responsible citizenship and ideal social values (Rose 1999b, Dean 2002) that recently were shown to exist in a Scandinavian context also (Michailakis and Schirmer 2010). The observation that patients are the targets of governing conduct is well known in the literature (see Armstrong 1983, Rose 1999a). However, what is significant about our findings, as we demonstrate later, is how GPs provide these patients with legitimacy through an interest in their social and personal profiles.

Our primary concern with the ‘making up’ (Hacking 1986) of a legitimate patient in the eyes of GPs leads to a focus on the constructive element of identities in institutions. We try to understand how institutional selves (Gubrium and Holstein 2001, Holstein and Gubrium 2000) rely heavily on the social context from which the categories in question derive.

## Data and methods

In order to gain a deeper insight in how GPs classify and recognise patients with MUS, 21 GPs were interviewed.^1^^,^^2^ All interviews were taped and transcribed. For ethical reasons the identities of the interviewees quoted in this article have been concealed. Participants in the study were made aware in advance that they would participate anonymously. In Denmark there are strict rules for processing and securing data. For example, we removed personal security numbers and last names from the interviews before sending them to transcription and we stored the data so only the research team could gain access to it. Besides these general rules for processing and storing the data and participant acceptance (based on descriptions of the research), no formal ethical approval to conduct a research project like this, is required in Denmark. We have, however, followed the guidelines given by the British Sociological Association on how to conduct research in an ethically responsible way.

### Individual and group interviews with GPs (N = 21)

We contacted the participating GPs for this study by randomly selecting and calling GPs across Denmark during their daily consultancy hours, between 08.00 and 09.00 am.^3^^,^^4^ Approximately half the GPs contacted declined to participate in the study either because they were too busy or because they found the research project irrelevant. The other GPs, however, decided to participate after being introduced to the study first on the telephone and later after having received written information on the study. The large number of GPs who declined to participate led us to reflect on our sample of those who agreed to participate. During our initial telephone contact and later interviews, it became clear that one dominant reason for GPs to participate was that patients with MUS were perceived as extraordinarily time consuming, medically challenging and often a demanding group to work with.

We could expect the GPs in our sample to have experience with MUS for two reasons. Firstly, citizens in Denmark can choose their own GP. Secondly, in an interview study with 41 patients with MUS we found that most change their GP if their current GP did not accommodate them as suffering individuals. Another common feature among the participating GPs was that they expressed the view that having MUS prevents individuals from fulfilling work-related obligations.

These reflections aside, conducting a research project based mainly on interview material produces certain possibilities and limitations for analysis. As such, the interview material should be seen as the result of ‘active encounters’ between the interviewer, with her theoretically motivated research agenda, and the interviewees, confronted with this agenda (Holstein and Gubrium 1997, Järvinen 2000). The interviews were semi-structured and maintained a focus on the interviewees’ perspective and subjective experience. We paid close attention to their involvement in the process by asking open-ended questions. In the actual interviews, for example, we often listened to longer disquisitions on the (problematic) social background of individuals with MUS that was believed to have a negative effect on their ability to get well. As a consequence, more discussion was elicited on this issue than we had initially prepared for. Thus, our research design was also adjusted during the interview process to better reflect issues of importance to the GPs.

The interviews with the GPs consisted of eight group interviews with 18 GPs in total and three individual interviews. Just as individual interviews can be seen as active encounters between interviewers and interviewees (Holstein and Gubrium 1997), group interviews also give special importance to the social context and the interactions among the interviewed participants in the story that is produced (Kitzinger 1994). In this case, the GPs could spur each other on to discuss various matters during the interview; this would result in discussions of central themes and sometimes parting in disagreement. The analysis has been thoughtfully conducted and the quotes presented in this article carefully selected, i.e., we have not presented quotes containing extreme opinions that are only shared by a few GPs. We have also been very attentive to shorter discussions among GPs that could be seen as examples of ‘spurring each other on’ and, as a result, have excluded these opinions from the analysis. In most cases, the group interviews with respectively three GPs (two groups) and two GPs (six groups) were conducted in a very similar fashion to the individual interviews with a question-and-answer structure. The GPs would give answers in turn and occasionally discuss the question posed among themselves. Systematic thematic readings of the data, however, did not reveal clear differences in the positions and attitudes of the GPs who were interviewed individually and those who participated in small groups. The three individual interviews conducted (with Peter, Martin and Monica) contained the same types of reflections as the groups in relation to the GPs’ explanation of the main problems of patients with MUS and the problem of labelling the patients’ complaints.

The eight group interviews each lasted approximately 1–2 hours, and the three individual interviews each lasted approximately 1 hour. All interviews, except one that was conducted at the university department, were conducted at the GPs’ workplaces. We began the analysis by reading the material in its entirety and listing the themes the interviewees talked about. We then systematically grouped the responses into themes and attitudes according to their relevance to our research questions. The following questions were used in our thematic reading: (i) What characterises this group of suffering people, according to the GPs? (ii) What labels and symbols are used to describe patients with MUS? (iii) What models of explanation are used to account for symptoms and complaints?

## The sick role and social problems

As discussed earlier, the classical literature has particularly focused on systems of classification and labelling and the effects of these systems on patients (Becker 1997, Foucault 1977, Goffman 1990a). This approach is important because it shows how any classification system, including that of medical diagnosis, can be seen as a social construct that reflects and is produced by the given social context. However, in our analysis we attempt to understand how GPs categorise symptoms and complaints and we examine the effects of GPs’ classifications.

A patient whose arm is crushed in a rolling machine in a bookbinding factory does not automatically play a legitimate sick role. Brian, one of the GPs, makes the following observation about an encounter with a former manual worker:

He got his arm stuck in a roll. The arm went all the way in. He crushed all his muscles and the arm swelled up to the size of a thigh, but he healed well; he didn’t undergo surgery and he regained full mobility. Now he turns up claiming that everything is wrong, but, you see, he can manage everything with his arm. He has moved apartment seven times; he has built five houses, but he claims that he cannot do anything at all.

As this doctor emphasises, the patient’s arm was completely healed. However, there is a discrepancy between the objective findings of the GP and the subjective complaints expressed by the patient. Similarly, another case reveals that it is not enough to have been involved in a traffic accident to be classified as legitimately sick when no physical evidence of damage is revealed in a computer tomography scan. As one of the GPs mentions, a ‘minor involvement in traffic accidents leads automatically to medical contact’ (Mary) or as another (Martin) explains:

When somebody crashes her car accidentally into something, which causes complaints of neck pain, then all others symptoms easily follow … manually you only find a little stiffness in the neck of the patient … the rest is just a retelling of the person’s own complaints … chronic pain, headache, concentration and memory problems, tinnitus, sexual difficulties and so on and so forth.

Instead, what the suffering individual must show to be recognised as a legitimate patient can depend on the GP’s emphasis on certain social background parameters. With MUS it is important to understand the categories through which GPs interpret patients’ complaints and how this classification proceeds from their perceptions of their patients’ (problematic) social background. As we will show, MUS cannot be separated from two important dimensions of social context: the individuals’ social problems and their (problematic) personality traits.

Despite differences in their responses, many GPs share the opinion that patients with MUS are ‘not born with a silver spoon in the mouth’, as one (Paul) explains. A violent family history, weak family ties and a lack of social resources can sometimes be enough to lend support to the sick role. Paul further elaborates that a young woman in her thirties who was married to a violent husband who ‘beats her nearly to death but looks like a dream for any mother-in-law but from whom she managed to escape; of course, she now has a condition’. This condition, Paul continues, lends the patient enough legitimacy to be declared ‘dysfunctional’. Paul ends his description by stating that such a patient is ‘somebody who, despite her young age, will never again return to the labour market … she just can’t manage it’. In addition to a violent husband who has changed a seemingly healthy woman into a ‘totally dysfunctional’ patient with unexplained pain symptoms, Brian explains that other social background factors, such as growing up under the wing of a distressed mother who was prescribed Valium, can justify medical attention.

Most often, GPs mention socially defined problems when characterising patients with MUS. These patients have few (if any) ‘resources’ (Brian), they are ‘non-educated’ (Diana), react to ‘problematic life circumstances … and have no motivation’ (Paul). They ‘don’t function’ (Michael), have ‘unacknowledged conflicts in their private life’ (Peter) and they are believed to have had a ‘problematic childhood’ (Susanne) in which they experienced ‘violence’ (Paul) and carried a ‘heavy load of desertion and neglect’ (Martin). A convincing picture emerges from our analysis of the interviews. As one of the GPs (Diana) explains, ‘these patients accommodate something social-wise’. In other words, patients with MUS are pictured as having many social problems due to their problematic upbringing, current social situation and social capacities.

To provide a more coherent description of GPs’ perceptions of patients with MUS, we quote Michael, who gives the following description of a typical encounter with this type of patient:

In the beginning, [patients with MUS] are very focused on the somatic problems … and you begin to examine their complaints. And you start some treatment for a bit of rheumatic disease and you give them some medicine to cure their pains … and you talk to them … and then it usually becomes apparent that they represent types of people that are disadvantaged.

Martin continues in the same line of thought:

They have experienced violent disturbances in their past … All kinds of things which possibly can go wrong in life, have somehow also turned out wrong for this group of people … at a moment in their life this social past is converted into somatic symptoms … pain, dizziness, headache, myalgia … classic somatic symptoms.

In our study, GPs appeared to construct a category of patients characterised by deviant social factors that differentiated them from ‘normal’ healthy people. The transformation of a suffering individual from a patient with somatic complaints into a patient with social problems, as shown in the above quotes, demonstrates a general pattern found in the interview material. Through this transformation, a legitimate institutional identity can be constructed despite the lack of physical evidence of any illness or physical disorder.

Broadly understood, to be a legitimate patient in the eyes of GPs is to be recognised as one who suffers (Cassell 2004). There is no doubt in our material that these doctors see patients with MUS as individuals who suffer, but it is less obvious *what* exactly they suffer from. Is it a malfunctioning body, a hypersensitive nerve system or poor genes? Or do they actually suffer from social problems related to a problematic childhood, an abusive partner or a bad economic situation? And, perhaps more importantly, can the latter – social problems of whatever kind – be united with the sick role? In other words, can you – medically speaking – suffer from social problems? Or do you need to suffer from more than social problems to be a legitimate patient?

## The sick role and problematic personality

Our analysis so far has shown that to be a legitimately sick patient, the complaints of a patient with MUS must be explained with reference to particular social aspects of that person’s life. But as the question just posed suggests, social problems might not be enough of a burden to qualify. The legitimacy of a patient – or the ‘permission to be ill’ (Nettleton 2006: 1167) – only becomes truly manifest, as we will show below, if social background parameters are combined with the personality of patients with MUS in the classification process.

As Monica explains, this patient group’s symptoms stem from a ‘combination of physical things … and a personal shortcoming’. Many GPs express the opinion that patients with MUS suffer from some sort of physical pain, but few discuss pain using a medical model that focuses on, for example, what kind of new diseases the pain might reflect. Of course, GPs regularly use medical terminology in their descriptions. For example, they say patients with MUS are ‘symptoms producers’ (Brian), are ‘chronically tired’ (Paul), have a ‘pre-morbid psyche’, have ‘a sensitive nervous system’ (Monica) and ‘have a dysfunctional disorder’ (Michelle). However, GPs typically relate these vaguely medical descriptions to the patients’ problematic social backgrounds and deviant personalities regardless of their physical complaints.

In our data, GPs often provide explanatory models for ‘somatisation’ that are grounded not only in physical distress and social problems but also in the specific personality types of patients with MUS. This awareness of the problematic personality traits of patients with MUS may be another way that doctors shift their attention away from physical complaints that cannot be observed and towards other aspects of these patients’ situations. Susanne, for example, explains that:

it is very often patients who have a certain type of personality. They don’t necessarily have a low social status or just moderate abilities, but it is presumably a question of personality types.

And Brian adds that ‘somebody just happens to be bowled over and just lies there … it has a lot to do with personality’.

As we read through the interviews, it became clear that, as Susanne points out, ‘there are certain kinds of personality types who easily turn into this kind of a patient’. Despite the fact that a social group such as patients with MUS can be seen as an ‘ill-defined, fuzzy, practical and symbolic construct’ (Jenkins 2010: 13), these individuals transform into a clinical workable whole by GPs’ ascriptions of problematic personalities. Here is another example illustrating this phenomenon, where two participants discuss in some detail the personality of patients with MUS:

Brian: Even on days when I’m full of positive energy … in other words my tank is totally full … then I enter the waiting room and there – Bang – there she is, now she sits there again … it is heavy.

Ann: They are infectious.

Brian: Yes, they are indeed infectious, aren’t they?

Ann: I used to say that one can feel when some of them show up at the clinic, how their energy seems to be withdrawn and tugged out from their big toe and is spilled out on the floor, don’t you think? One can feel them down there; there is no energy present, no drive at all.

Ann’s acknowledgement of the patients as infectious is supplemented by another female GP, Diana, who explains, ‘When they fall off the treadmill, they can’t get on again’. Peter has a similar observation concerning personality in relation to coping capabilities: ‘They see half-empty glasses’. Or, as Brian says, ‘They see problems instead of tasks to fulfil’. Lisa, a female GP, believes that ‘a lot of them have a wandering personality, which gets them into all kinds of trouble’. What might, from an outsider’s point of view, seem to be very offensive images of a particular patient group is, however, also supplemented by more emotionally loaded descriptions and verbs. For example, Diana and others point out that patients with MUS are very ‘sensitive’, they are ‘tired’ (Michelle), ‘they don’t display any happiness; they are rather joyless’ (Brian), ‘they have a low threshold of frustration … a low threshold for adversity, stress and demands’ (Susanne), their ‘lives simply hurt’ (Martin) and finally, Michael concludes that they are ‘inept at living … whiners … pitiable people’.

Despite the variety of the metaphors used by the GPs ranging from lay to professional terminology, there is a common pattern in their utterances: the metaphors’ ability to produce associations that characterise a certain type of personality. Together, the list of (problematic) social background parameters and the different (problematic) personality types make up a common pattern that patients with MUS can be fitted into. The GPs are then able to recognise a familiar pattern in the patients’ complaints, a pattern that enables the GPs to proceed in the emerging diagnostic process and reach a sort of final point in the process that forms the basis for further intervention.

GPs’ professional identities as doctors depend on their ability to construct patients out of people complaining about pain. They must provide a diagnostic trajectory, which may result in an improvement of the patients’ situation. That is, not only do patients with MUS need to be conceptualised as legitimate in their complaints but also the GPs are in need of legitimate patients in the encounter to be judged as credible or infallible professionals (Jutel 2010). According to our findings, GPs discover in patients with MUS some kind of fundamental human weaknesses on both a social and personal level, which can constitute a useful pattern for further interventions. When GPs focus on the social background and personality traits of patients with MUS, they are able to treat individuals with MUS as legitimate patients.

## Discussion

In this article we have explored the negotiation of a legitimate sick role for patients with MUS in primary care. We have analysed what it takes from the GPs’ perspective for a sick role to be seen as legitimate. Our findings show that the GPs’ evaluation of the legitimacy of individuals with MUS, who are suffering and therefore unable to work and take on daily duties, relates first and foremost to an assessment of the social background and particular personality type of patients with MUS. When a problematic background combines with a problematic personality as a series of rather distinct elements, GPs can accept patients’ medical legitimacy as suffering individuals and try to accommodate their particular complaints.

Other studies on MUS in medical practice have analysed patients’ perspectives (Dumit 2006, Nettleton 2006, Werner and Malterud 2003), primarily examining how patients experience their encounters with GPs and how they work to be understood and taken seriously as patients. However, little research has been done on how the sick role of patients with MUS is negotiated from the doctors’ point of view or on the explanatory models used by doctors in this process. This study attempts to shed light on these problems by focusing on the external side of the identity-formation process in which the sick role is negotiated; that is, how formal classification practices among doctors produce legitimate patients.

The lack of patient voices might appear to be a weakness in the article. Other studies, for example, show how patients do not trust doctors with discussions of emotional aspects of their problems and instead choose to hide those aspects behind somatic symptoms (Peters *et al.* 2008) or how doctors fail to respond to hints of the patients’ desire for emotional support (Salmon *et al.* 2008). We have, however, deliberately left patients’ voices out because our theoretical perspective favours ‘the others’– the external validation – in the social process of identity work (Gubrium and Holstein 2001, Mead 1934). The role of a legitimate patient, in other words, is not a role a suffering person can just take – this sick role is a position that can be given to an individual by doctors only if the suffering individual’s story and situation resemble, in GPs’ eyes, the story and situation of paradigmatic patients with MUS. Legitimation is thus an institutionalised matter. Patients with MUS and their specific illnesses are in this way assembled and legitimised as much in virtue of their own experience of illness as in virtue of GPs’ perceptions of what it takes to be a legitimate patient.

The GPs’ explanation of legitimacy in the medical encounter turns patients with MUS into objects of recognition (Foucault 1977). As noted by Atkinson (1995: 149), a general feature of modern medicine seems to be ‘the dislocation of the case from the patient’s bedside’ and indeed from the patient’s physical presence. Our analysis of how GPs classify and recognise patients with MUS demonstrates how the patient is transmuted into an object of careful inspection and discourse that is both set outside a traditional biomedical vocabulary and is physically dislocated from the patient. The lack of a traditional medical diagnosis of patients with MUS leads GPs to create a new kind of category, a social diagnosis, which resembles a clinical diagnosis in its function as an explanatory model for further intervention but is different from a clinical diagnosis in its lack of attention to pathological components. A possible effect of social diagnosis-making may be that GPs in some cases regard the suffering of patients with MUS as simply manifestations of the social.

GPs’ discussions of the sick role show that a legitimate patient identity is closely tied to social responsibilities and to the performance of certain societal obligations. We now conclude by suggesting how the current sick role might be intimately connected to social obligations, as proposed in the work of Parsons (1951: 1978). The socio-political treatment of individuals by GPs is largely infected by their role as professional experts. When framing or establishing a legitimate patient, GPs not only stabilise their own professional identity as doctors; they might also take into account general societal norms about the obligation to participate in the labour market if one doesn’t have a medically explainable physical or psychological defect. It becomes part of the GP’s job to help patients with MUS to be responsible citizens in relation to socioeconomic and political demands of labouring, such as obligations to wake up in the morning, to hold down a job and to manage the daily tasks in one’s life. In other words, medicine could be seen as a profession that is engaged in translating and rearticulating contemporary norms in society concerning what it takes to be a responsible citizen in modern western societies (Dean 1998, 2002, Michailakis and Schirmer 2010). In the words of one GP, ‘The trick is to help these patients to be able to take on a job’, a sentiment echoed by other GPs. The GPs’ evaluation of a patient’s legitimacy, in other words, relates, perhaps primarily, to an evaluation of the possibility that the individual could return to the labour market. This evaluation seems to be very focused on the social background and personality types of patients with MUS. The evaluation of legitimate patients, i.e., individuals who are unable to support themselves at a given moment, might be intimately connected to what is perceived to be the social obligations of citizens.

How to respond to patients suffering with MUS, however, is one of the fundamental dilemmas of contemporary medical practice in primary care (Wainwright *et al.* 2006), which leaves the GPs adrift in an uncertain domain. As Griffiths *et al.* (2005) have shown, GPs prefer problem formulations, such as diagnoses, that have simple solutions as a way to create order in the midst of the chaos and confusion that their patients present. On one hand, doctors might find themselves frustrated by their inability to come up with a clear-cut diagnosis in medical encounters with patients classified as suffering from MUS. On the other hand, this particular patient group is in need of a diagnosis to validate their diffuse symptoms, which pervade most aspects of their lives.

Our findings demonstrate that GPs are prepared to set aside the traditional search for objective findings to confirm the subjective complaints of patients with MUS. This happens by constructing and negotiating a sick role even when there is a lack of a clear-cut medical diagnosis and it is difficult to label a particular illness. As Parsons (1951) argued, this process gives patients access to the sick role and gives medicine its power to legitimise or construct illness. But this construction of the sick role also, perhaps unintentionally, determines that patients with MUS are individuals who suffer from a combination of social problems and problematic personality traits. To be a legitimate patient, then, does not come without side effects. GPs become co-producers of novel sick roles that might have consequences for the everyday life of these people, including their social relations with their family, employers and the welfare state in general.

## Footnotes

1 This research is part of a larger study consisting of a national survey of welfare officers working in the area of sickness benefits in Denmark’s 98 municipalities, 41 qualitative individual interviews with people with MUS, 15 group interviews with welfare officers (*N* = 52) and three individual interviews and eight group interviews with GPs (*N* = 21) (Mik-Meyer 2010, 2011).

2 We initially planned to conduct only group interviews with GPs. However, in three cases, only one GP was present (the others were either sick or too busy to participate as promised). We have, however, decided to include both the three individual interviews and our group interviews in our analysis.

3 This research project’s methodological design, data collection and processing were conducted solely by Nanna Mik-Meyer and a research assistant, but to harmonise the writing style in this article, we have decided to write ‘we’ throughout our discussions in this methodological section.

4 Unlike in other countries such as the UK, it is still possible in Denmark to get in personal phone contact with GPs during the daily consultancy times.
